# Viewpoint on *Emv2*, the onlhy endogenous ecotropic murine leukemia virus of C57BL/6 mice

**DOI:** 10.1186/1742-4690-9-25

**Published:** 2012-03-22

**Authors:** Christine A Kozak

**Affiliations:** 1 Laboratory of Molecular Microbiology, National Institute of Allergy and Infectious Diseases, Bldg. 4, Room 329, 4 Center Drive MSC 0460, Bethesda, MD 20892-0460, USA

## Abstract

Here I comment on the articles by Lee and colleagues (Retrovirology 2011, 8:82) and Lee and Cho (Retrovirology 2012, 9:23) dealing with an endogenous ecotropic mouse leukemia virus found in C57BL mice.

## Viewpoint

The paper by Lee and colleagues [1] and their additional comment on this work [2] argue that C57BL strain mice carry an endogenous ecotropic murine leukemia virus (E-MLV) with intact coding potential that may be distinct from the previously described *Emv2*. However, a review of the published literature and analysis of the sequenced genome do not support this view, as pointed out by Young and colleagues [3]. It is apparent that some confusion has resulted from the fact that the sequenced mouse genome is poorly annotated for retroviral sequences, from observations that the *Emv2 *provirus is replication incompetent, and because the original map location of *Emv2 *on the Chromosome (Chr) 8 linkage map is not precisely identical to its location on the database map Build. It is worthwhile elaborating on several points relevant to this issue: the fact that mice carrying only *Emv2 *can, in fact, produce infectious virus, and the evidence supporting the map location of this functional E-MLV provirus on distal Chromosome (Chr) 8 of C57BL mice.

Three different subtypes of E-MuLVs have been found in inbred and wild house mice, but only the AKV subtype is present in the common strains of laboratory mice. The different strains carry different numbers of these *Emv *proviruses inserted in different chromosomal locations [4,5]; this explains the detection of *Emv2 *sequences in many inbred strains by Lee *et al. *[1] using primers that are E-MuLV-, but not locus-specific, and also explains the presence of related sequences in *M. m. molossinus*, a wild mouse species previously shown to carry AKV-type *Emv *loci [6].

As pointed out by Young and colleagues [3], Southern blot analysis of C57BL DNA and examination of its sequenced genome identify one and only one ecotropic MLV provirus in this mouse strain that is found in the BAC RP23-214K5 (GenBank No. AC158362, position 97057-105700); this full-length provirus, *Emv2*, is 99.5% identical to the infectious AKV MLV derived from *Emv11 *(J01998). A *pol *defect compromises the ability of the *Emv2 *sequence to produce infectious virus when expressed in transfected cells [7]. However, *Emv2 *can and does produce E-MLV in vivo in aging C57BL mice [8] and cultured cells from these mice can also be induced by halogenated pyrimidines to produce ecotropic virus, although inefficiently compared to cells carrying other *Emv *proviruses [5,9]. This induction process is, however, efficient enough that 40% of individual mice from backcrosses between C57BL mice and E-MLV negative mice could be induced to produce infectious virus, which is consistent with inheritance of a single functional though poorly expressed provirus [5]. The fact that this provirus produces E-MLV in cultured cells relatively soon after induction suggests that the generation of infectious virus in mice carrying *Emv2 *is a relatively common event, and likely results from recombination because expression is significantly enhanced in mice carrying *Emv2 *along with the full-length but envelope-defective *Emv1 *[7,9,10]. The detection of virus expression by Lee *et al. *in C57BL mice [1] does not therefore warrant postulating the presence of a second locus.

The BAC carrying the C57BL E-MuLV maps to distal Chr 8, and it should also be pointed out that the chromosomal map location of *Emv2 *was initially reported decades ago through analysis of C57BL backcross mice for the inherited ability to produce ecotropic virus after induction [5]. In multipoint genetic crosses, *Emv2 *(then termed *Bv*) was positioned as being 23.5+/-4.2 cM from the locus *Got2 *in two crosses, 25.9+/-4.8 cM from *Ces1c *(formerly *Es1*), and showed no linkage with the more proximal Chr 8 marker *Gsr *(formerly *Gr1*). The recombinational distances between the reference genes are consistent with their gene order and chromosomal positions in the sequenced genome, and with the map locations for these genes given by Mouse Genome Informatics (URL = http://www.informatics.jax.org/). Other studies typed the CXB and BXH recombinant inbred (RI) lines for the *Emv2 *sequence and for its expression, and the cumulative marker typing information from these RI strains also positioned *Emv2 *on distal Chr 8 [9,11], as did analysis of *Emv2 *complementation of *Emv1 *in backcross mice [12]. This proviral locus was routinely included on maps of retroviral loci and on the developing linkage map of Chr 8 [13]. The *Emv2 *map locations defined by these various linkage analyses were determined with genetic methods that are crude by today's standards; these methods placed *Emv2 *within a 95% confidence interval of 8.4-10.7 cM on distal Chr 8, an interval defined by the frequency of recombinational events rather than by an actual physical distance. The data derived from these recombination-based studies are not, however, inconsistent with the position of this *Emv *in the sequenced genome.

## Conclusions

It is clear from published work that the provirus identified in the Lee *et al. *study [1] is, in fact, *Emv2*, and no other tests are needed to accept this conclusion. Investigators should bear in mind that annotation of endogenous retroviral sequences in the sequenced mouse genome is incomplete, and that some of the available linkage maps represent best-guess consensus maps that attempt to incorporate data from disparate sources. Reliance on the information in databases does not therefore guarantee accurate characterization of individual ERVs, and it is therefore important to seek out primary sources for supporting documentation.

## Competing interests

The author declares that they have no competing interests.
